# Multiplexed immunolabelling of cancer using bioconjugated plasmonic gold–silver alloy nanoparticles†

**DOI:** 10.1039/d4na00052h

**Published:** 2024-07-03

**Authors:** Cécile Darviot, Bryan Gosselin, Flavie Martin, Sergiy Patskovsky, Ivan Jabin, Gilles Bruylants, Dominique Trudel, Michel Meunier

**Affiliations:** a Polytechnique Montréal Montréal Canada michel.meunier@polymtl.ca; b Centre Hospitalier de l′Université de Montréal Montréal Canada; c Université Libre de Bruxelles, LCO Bruxelles Belgium; d Université Libre de Bruxelles, EMNS Bruxelles Belgium

## Abstract

Reliable protein detection methods are vital for advancing biological research and medical diagnostics. While immunohistochemistry and immunofluorescence are commonly employed, their limitations underscore the necessity for alternative approaches. This study introduces immunoplasmonic labelling, utilizing plasmonic nanoparticles (NPs), specifically designed gold and gold–silver alloy NPs (Au:Ag NPs), for multiplexed and quantitative protein detection. These NPs, when coupled with antibodies targeting proteins of interest, enable accurate counting and evaluation of protein expression levels while overcoming issues such as autofluorescence. In this study, we compare two nanoparticle functionalization strategies—one coating based on thiolated PEG and one coating based on calix[4]arenes—on gold and gold–silver alloy nanoparticles of varying sizes. Overall results tend to demonstrate a greater versatility for the calix[4]arene-based coating. With this coating and using the classical EDC/sulfo-NHS cross-linking procedure, we also demonstrate the successful multiplexed immunolabelling of Her2, CD44, and EpCAM in breast cancer cell lines (SK-BR-3 and MDA-MB-231). Furthermore, we introduce a user-friendly software for automatic NP detection and classification by colour, providing a promising proof-of-concept for the practical application of immunoplasmonic techniques in the quantitative analysis of biopsies in the clinical setting.

## Abstract 2

Multiplexed and quantitative detection of analytes in cancer samples using specifically designed bioconjugated gold–silver alloy nanoparticles (NPs), a simple microscope adapter, and user-friendly software for the automated classification of NPs.

## Graphical abstract

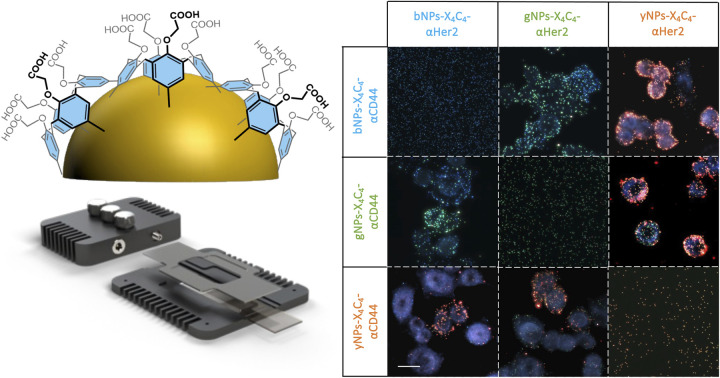

## Introduction

Reliable protein detection methods are crucial for advancing biological research and medical diagnostics. In particular, quantification of protein expression levels in biopsies is usually performed with immunohistochemistry (IHC), a semi-quantitative technique that often lacks the desired accuracy.^1^ While immunofluorescence (IF) stands out as a promising alternative in research due to its impressive multiplexing capability and resolution,^2^ precise quantification remains challenging. Furthermore, the overall process is time-consuming, and the presence of autofluorescence in certain tissues limits the widespread adoption of IF in clinical facilities.^3^ To address this challenge, researchers have been investigating the use of plasmonic nanoparticles (NPs) as protein tags, enabling multiplexed and quantitative detection of analytes in biological samples.^4,5^ Compared to fluorophores, individual plasmonic NPs exhibit a strong signal through light scattering, which allows detection at the single NP level. Moreover, due to their excellent stability, plasmonic NPs do not suffer from photo-bleaching. In particular, we have reported specifically designed gold–silver alloy NPs (Au:Ag NPs) displaying optimal optical properties for such use.^6^ When coupled with antibodies (Abs) targeting proteins of interest, these NPs can be used to target cells expressing these antigens. Using darkfield microscopy, the number of plasmonic particles bound to the cells can be precisely counted, allowing the evaluation of protein expression levels.^4^ Termed immunoplasmonic (IP) labeling, this method provides reliable results unaffected by autofluorescence and does not require extensive intensity calibration, offering a promising avenue for accurate protein expression level quantification.

Earlier research has included refining a darkfield microscopy approach to optimize the contrast of nanoparticles on cell membranes. This method, entitled side-illumination microscopy, relies on the lateral illumination of the sample with Red-Green-Blue (RGB) Light Emitting Diodes (LEDs). Collecting the signal in inverted or upright mode produces the expected darkfield effect while reducing the signal from the cell membranes and can be performed with a simple RGB camera.^7^ Notably, we have pinpointed the most effective NPs for protein detection by selecting nanoparticles with Localized Surface Plasmon Resonance (LSPR) bands that align with our lateral illumination diode wavelengths and present high scattering intensity. Achieving such combinations requires the use of NPs with diverse sizes and compositions.

One key challenge in using NPs as protein reporters is to ensure precise control over the grafting of antibodies, which is critical to achieving high specificity of interaction with the target cells. This step should, however, not lead to particle aggregation, which would alter the NP plasmonic properties. Typically, bioconjugated nanoparticles find application in lateral flow assays,^8–10^ immunoblotting,^11^ or immunoprecipitation assays,^12–15^ where stringent control over the level of aggregation is not imperative. However, in IP labelling, where each individual NP is detected under the microscope, maintaining individual nanoparticles is of utmost importance to retain their original colour. Moreover, as our technique involves a variety of NPs, selecting a bioconjugation method that performs uniformly on all the NPs is essential to avoid biases arising from differences in functionalization densities. A common way to conjugate proteins to NPs involves the EDC/sulfo-NHS cross-linking procedure, which requires the presence of carboxyl groups on the surface of the NPs. The use of thiolated PEG-COOH on gold^16–18^ and silver^19,20^ NPs as a precursor for antibody grafting has been extensively studied and documented. However, some studies indicate that the thiolation of silver NPs might be challenging for some applications.^21^ Given our approach involves gold–silver alloy NPs as well as gold NPs, we have also investigated the use of a calix[4]arene-based coating obtained through the reduction of a calix[4]arene-tetradiazonium salt.^22,23^ Indeed, due to its extreme stability, such coating has shown superior performance in protein bioconjugation on silver surfaces compared to traditional thiolation methods,^24^ suggesting that this strategy would be efficient for gold–silver alloy nanoparticles.

Our study aims to explore and compare two identified strategies, one involving a thiolated PEG_5kDa_ coating and the other a calix[4]arene-based coating, for bioconjugation of nanoparticles of various sizes and compositions and their use as protein reporters. We employed 100 nm AuNPs, 50 : 50 Au : Ag alloy NPs measuring 63 nm, and 10 : 90 Au : Ag alloy NPs measuring 50 nm. These specific NP sizes and compositions were selected based on the position of their scattering peaks and to ensure similar light scattering intensities. For conciseness purposes, we will refer to the NPs using their corresponding scattered colours: the 100 nm AuNPs as yellow NPs (yNPs), the 63 nm 50 : 50 Au : Ag NPs as green NPs (gNPs) and the 50 nm 10 : 90 Au : Ag NPs as blue NPs (bNPs). Our research outcomes encompass successful multiplexed immunolabelling of Her2, CD44, and EpCAM in the SK-BR-3 and MDA-MB-231 breast cancer cell lines. Additionally, we have developed a user-friendly software capable of automatically detecting and classifying nanoparticles based on their distinct colours. These advancements serve as a promising proof-of-concept for the practical application of the immunoplasmonic technique in quantitative biopsy analysis.

## Materials and methods

### Nanomaterials and supplies

The 100 nm, 60 nm gold and 60 nm silver nanoparticles were purchased at Nanocomposix, San Diego. The alloy nanoparticles of various sizes and compositions were synthesized according to the previously reported method.^6^ First, gold seeds of about 13 nm in diameter are synthesized using the Turkevich method. Then, 2 to 3 growth steps are performed, using the method described at length in the ESI.† The HS-PEG_5kDa_-COOH was purchased at Nanocs. The X_4_C_4_ was synthesized according to the previously reported method.^25^ The synthesis of calix[4]arene-tetraacid tetradiazonium salt X_4_C_4_ was achieved according to the literature^26^ in four steps (*i.e. ipso*-tetra-nitration, reduction of the four nitro groups, ester hydrolysis and diazotation in 57% overall yield) from commercially available 4-*t*-butylcalix[4]arene-tetraacetic acid tetraethyl ester (Eburon Organics). Note however that the reduction of the nitro groups of the intermediate tetra-nitro derivative was achieved in 94% yield through hydrogenation (H_2_, Pd/C) and not by using SnCl_2_, as it was previously described. The NPs were functionalized with αCD44 antibodies (Hermes1, MA4400, ThermoFisher), αHer2 antibodies (ICR12, ab11710 and EPR19547-12, ab214275 from Abcam), and αEpCAM antibodies (ab71916, Abcam).

### Nanoparticles characterization

Transmission Electron Microscopy images of citrate nanoparticles were taken with a JEM-2100 instrument from JEOL. Briefly, the NPs were centrifugated and resuspended in ethanol. The NPs were then placed onto a copper grid coated with a thin carbon film for electron microscopy observation. All obtained NPs were also characterized with the UV-visible spectrometer Epoch and the hydrodynamic diameters were measured using the Zetasizer Pro Blue instrument from Malvern Panalytical (Malvern, U.K.) in an Ultra-Micro Quartz Cell (ZEN2112). Attenuated total reflection Fourier-transform infrared (ATR-FTIR) spectra were recorded at 20 °C on a Shimadzu QATR-S FTIR spectrophotometer. The nanoparticles were centrifugated, and 2 μL of the pellet were deposited on the diamond. Water was removed with a flow of nitrogen gas. Data were processed and analyzed using the instrument software by correcting the baseline, setting apodization at 10 cm^−1^, and normalizing on the most intense signal. The bioconjugation of NPs was verified using a homemade lateral flow assay. Briefly, protein A/G at 1 mg mL^−1^ was deposited with a syringe on a nitrocellulose membrane and dried at 40 °C for a minimum of 2 h. Then the absorbent pad was affixed to the membrane and the membrane was cut into 4 mm strips. The conjugation test is performed by diluting 1–2 μL of NPs (∼5 × 10^10^ NPs/mL) in 40 mL of PBST in a well from a 96-well plate. The test is then immersed vertically in the well, allowing the nanoparticles to migrate along the membrane by capillary action. After all the liquid has passed through the membrane (approximately 5–10 min), the test is ready for interpretation.

### Nanoparticles functionalization

For the PEGylation of NPs, the HS-PEG_5kDa_-COOH was first reduced using TCEP by adding 10% of TCEP·HCl (0.5 M in dH_2_O) in a solution of SH-PEG_5kDa_-COOH (10 mg mL^−1^ in dH_2_O) for 15 min. The TCEP was then filtered twice using 3 kDa centrifugal filters. 300 μL of the activated SH-PEG_5kDa_-COOH was then added to 10 mL of citrate-capped NPs in glass vials and left overnight at room temperature (RT), under stirring. The next day, excess PEG was removed through 3 centrifugation cycles and the PEGylated NPs were resuspended in milliQ water.

For the functionalization of gNPs, yNPs (or bNPs) with X_4_C_4_, 150 μL (100 μL in the case of bNPs) of NaBH_4_ at 100 mM was added to 10 mL of citrate-capped NPs in glass vials. Then, 2 × 500 μL (1 × 500 μL in the case of bNPs) of X_4_C_4_ at 5 mM were added drop by drop in the solution under stirring. The reaction mixture was stirred overnight at RT and then 20 μL of NaOH at 1 M was added under stirring. The NPs were then rinsed through three centrifugation cycles with NaOH 5 mM. At the last cycle, the NPs-X_4_C_4_ were resuspended in Milli-Q water.

### Nanoparticles bioconjugation

Two slightly different procedures were followed for the NPs-X_4_C_4_ or the NPs-PEG_5kDa_-COOH as the attempts to homogenize the protocols were leading to aggregations of NPs or inefficient antibody grafting. In a 1.5 mL VWR centrifuge tube, 150 μL of NPs-X_4_C_4_ (∼5 × 10^10^ NPs/mL) was added. Then, 15 μL MES (100 mM, pH = 5.8), 15 μL EDC·HCl (6 mM, 1.15 mg mL^−1^ in milliQ H_2_O) and 15 μL Sulfo-NHS (10 mM, 2.17 mg mL^−1^). The activation step was carried out for 1 hour and then, the centrifuge tube was filled to 0.75 mL with MilliQ H_2_O. The centrifuge tube was centrifugated once (12 min, 3000 g) at room temperature. The supernatant was discarded, and the pellet was resuspended in 150 μL phosphate buffer (5 mM, pH = 7 or pH = 8). Then, 10 μL of antibodies (0.1 mg mL^−1^ in 5 mM PB pH 7 or 8) were added and the reaction mixture was stirred for 1 hour at room temperature. After 1 hour, 600 μL of a solution of 1% BSA, 0.1% Tween 20 in 5 mM PB pH 8. The centrifuge tube was stirred for 3–5 minutes and then, centrifuged for 12 min at 3000 g. Afterward, the supernatant was discarded, and NPs were resuspended in 1 mL of the solution 1% BSA, 0.1% Tween 20 in 5 mM PB pH = 8 and once again, centrifuged as before. At the last cycle, NPs were re-suspended in 150 μL of 0.1% Tween 20 in 5 mM phosphate buffer (pH = 7 or pH = 8). The resulting particles were stored at 4 °C.

The protocol for the PEGylated NPs differs slightly but shares the key steps. In a 1.5 mL centrifuge tube, 150 μL of NPs-PEG-COOH (∼5 × 10^10^ NPs/mL) were added. MilliQ was replaced by MES buffer (0.5 M) through the centrifugation cycle. The activation step was then carried out for 30 min by adding 13 μL of EDC·HCl (6 mM) and 7.4 μL of sulfo-NHS (10 mM). The excess EDC and sulfo-NHS were washed through a centrifugation cycle and the NPs were resuspended in 5 mM potassium phosphate (pH = 7) and 0.1% Tween 20. Then, 10 μL of antibodies (0.1 mg mL^−1^) were added and the reaction was carried out for one hour. The reaction was then quenched by adding 2 μL of hydroxylamine (50%) for 10 min. The NPs-PEG-Abs were then washed through 3 centrifugation cycles and resuspended in the 5 mM potassium phosphate (pH = 7) and 0.1% Tween 20. The resulting NPs-PEG-Ab were stored at 4 °C.

### Cell culture and immunolabelling

The breast cancer cells MDA-MB-231 (ATCC, HTB-26) were cultured in Dulbecco's Modified Eagle Medium (DMEM) while the SK-BR-3 (ATCC, HTB-30) cells were cultured in the recommended McCoy culture medium. Following standard practices, both media were supplemented with 10% FBS, and 1% P/S. We prepared the samples by seeding cells in 8 well plates with removable chambers (Ibidi) and letting the cells grow for 24 to 48 hours. Before immunolabelling, the cells were fixed with cold methanol/acetone (70/30%) for 10 min and rinsed with PBS. A one-hour blocking step was performed in (PBS, Tween 20 0.1%, 1% BSA) and the samples were rinsed with PBS before protein labelling with the method of interest. For immunofluorescence, two times 1 h incubations were performed with a dilution of 1 : 200 and 1 : 500 respectively for the primary and secondary antibodies. For the immunoplasmonics method, the NPs were incubated for one hour with the cells. After immunolabelling, the cells were rinsed 3 times with PBS and 0.1% Tween 20.

### Cell imaging

The samples were imaged on an inverted Ti-Eclipse microscope (Nikon) equipped with a lateral illumination adapter for the darkfield mode, as reported previously.^7^ The images were taken with a colour camera (pandas4.2, PCO) with fixed parameters. The images were taken using alternatively a 40X-0.6 air objective or a 60X-1.1 N.A. immersion oil objective, and the sample was scanned along the *z*-axis to capture all the NPs present on the cell membranes. The acquired slices were then stacked and projected onto a single picture that was later used for the analysis.

## Results

### Nanoparticles coating with either a thiolated PEG_5kDa_ or a calix[4]arene

**Scheme 1:**
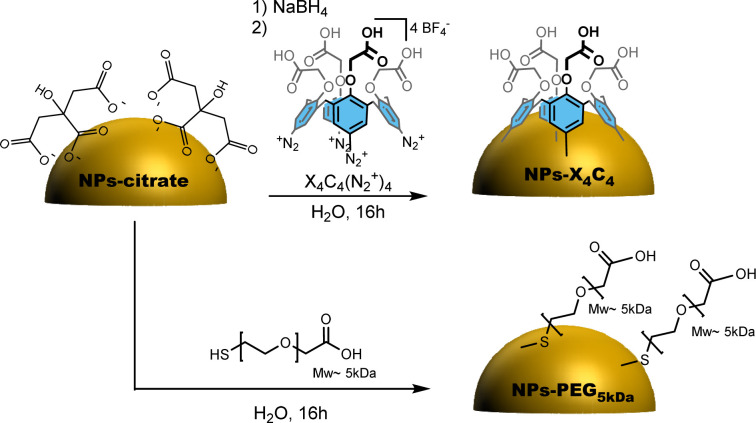
Preparation of NPs-PEG_5kDa_ and NPs-X_4_C_4_.

The citrate-stabilized alloy NPs were first synthesized using a previously reported procedure.^6^ TEM and EDS measurements showed that we achieved highly monodisperse 50 nm 15 : 85 Au : Ag NPs displaying a blue colour, and 63 nm 43 : 57 Au : Ag NPs displaying a green colour as shown in Fig. S1,† displaying the TEM and UV-vis characterization of these NPs. They were then functionalized either through chemisorption of a thiolated PEG, *i.e.* HS-PEG_5kDa_-COOH, or reductive grafting of calix[4]tetra-diazonium salt X_4_C_4_(N_2_^+^)_4_ (Scheme 1). For conciseness, in the rest of the paper, we will refer to the two types of coating as PEG_5kDa_ and X_4_C_4_ coatings, respectively. The resulting NPs-PEG_5kDa_ and NPs-X_4_C_4_ were characterized through Dynamic Light Scattering (DLS), UV-vis spectroscopy, and Fourier-Transform InfraRed (FTIR) spectroscopy. For all the NPs-X_4_C_4_, we observed a mean increase in the hydrodynamic diameter of 15 nm (Table 1). For the PEG_5kDa_ coating, we measured an increase in the hydrodynamic diameter of 37 nm for the yNPs and 20 nm for the bNPs and gNPs.

**Table 1:** Typical hydrodynamic diameters of the NPs measured by DLS

	Hydrodynamic diameter (nm)		
	Citrate	X_4_C_4_	PEG_5kDa_
bNPs (Au : Ag 10 : 90 50 nm)	50.8 ± 0.3	68.1 ± 0.6	71.2 ± 0.4
gNPs (Au : Ag 50 : 50 63 nm)	70.7 ± 1.0	86.0 ± 0.6	90.8 ± 1.1
yNPs (Au 100 nm)	98.8 ± 0.7	114.4 ± 0.7	136.4 ± 0.3

**Fig. 1:**
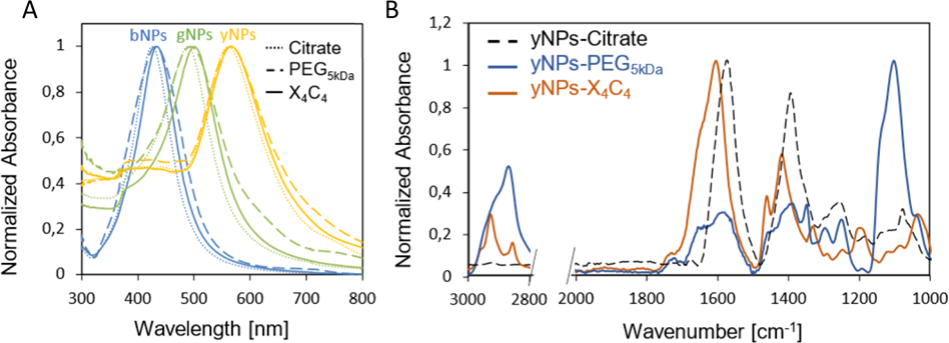
(A) UV-vis spectra of the various NPs before and after functionalization with HS-PEG_5kDa_-COOH or X_4_C_4_; (B) FTIR spectra for yNPs with various surfactants.

In addition, the functionalization led to a red shift of the plasmon peak position of 3, 9, and 12 nm for the X_4_C_4_ and 2, 5, and 7 nm for the PEG_5kDa_ coatings of yNPs, gNPs, and bNPs, respectively (Fig. 1A). These shifts in the extinction spectra can be attributed to changes in the surrounding environment of the nanoparticles, indicating a modification of their organic coating. The extent of the shift correlates with the intrinsic properties of the NPs, the thickness of the coating corona and the effective refractive index of the coating. The PEG_5kDa_ a layer is mostly swollen by water and, therefore, its effective refractive index is relatively close to that of water. In contrast, the X_4_C_4_ coating should lead to a significantly higher refractive index than water due to the presence of the hydrophobic polyaromatic macrocycles. Therefore, despite being thinner, the X_4_C_4_ coating induces a much greater peak shift than the PEG_5kDa_ layer. A certain broadening of the LSPR band was also observed for all NPs-PEG_5kDa_, indicating a higher aggregation level or a higher disparity in surface coating with this ligand (Fig. 1A).

The characterization of the resulting NPs-X_4_C_4_ using FTIR spectroscopy confirmed the grafting of calix[4]arene X_4_C_4_, with characteristic bands at 1600 and 1450 cm^−1^ corresponding to COO^−^ stretching and aromatic ring stretching, respectively.^27^ The FTIR spectrum of NPs-PEG_5kDa_ is dominated by bands attributed to C–O–C asymmetrical stretching (1110 cm^−1^) and C–H(CH_2_) stretching (2840–3000 cm^−1^) from the long polymer chain. The spectra obtained for yNPs are shown in Fig. 1B, while comparisons for all types of NPs are available in the ESI (Fig. S2).† It is noteworthy that characteristic bands originating from citrate, such as those at 1590 and 1400 cm^−1^ for COO^−^ asymmetrical and symmetrical stretching, were no longer observed after functionalization, confirming the efficiency of the ligands exchange process.

**Fig. 2:**
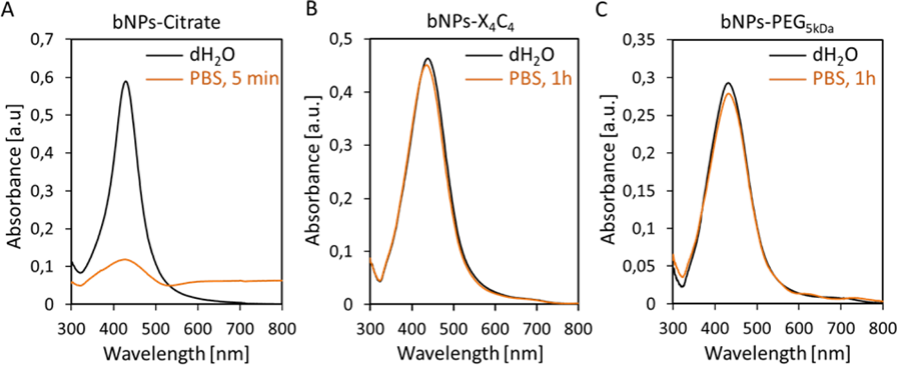
UV-vis spectra of bNPs (Au : Ag 10 : 90 50 nm) in PBS: (A) citrate coating; (B) X_4_C_4_ coating; (C) PEG_5kDa_ coating.

The colloidal stability of NPs-PEG_5kDa_ and NPs-X_4_C_4_ was evaluated in saline buffer (PBS 1×) and compared to that of NPs-citrate. The results obtained for the bNPs are presented in Fig. 2 (see Fig. S3† for gNPs and yNPs). As shown in Fig. 2A, a significant modification of the UV-vis spectrum was observed for citrate-capped NPs, indicating NPs aggregation and dissolution. Indeed, citrate-capped NPs are only stabilized through electrostatic repulsion between the negatively charged citrate molecules. When the ionic strength is increased by the addition of PBS, the electrostatic repulsion vanishes leading to the aggregation of the NPs driven by the attractive van der Waals forces. In contrast, the coatings based on calix[4]arene and thiolated PEG ligands led to stable colloids in PBS, validating these functionalization strategies (Fig. 2B and C). Regarding the PEGylated NPs, although the yNPs-PEG_5kDa_ remained stable for months (at RT in the dark), both bNPs-PEG_5kDa_ and gNPs-PEG_5kDa_ started to show signs of instability after a few weeks (decreasing OD, adsorption on tubes). Conversely, no significant loss was observed for all NPs-X_4_C_4_, highlighting their long-term storage stability which stands as a clear advantage for practical applications in biopsy diagnostics in clinical settings.

### Bioconjugation using covalent bonding or adsorption

**Fig. 3:**
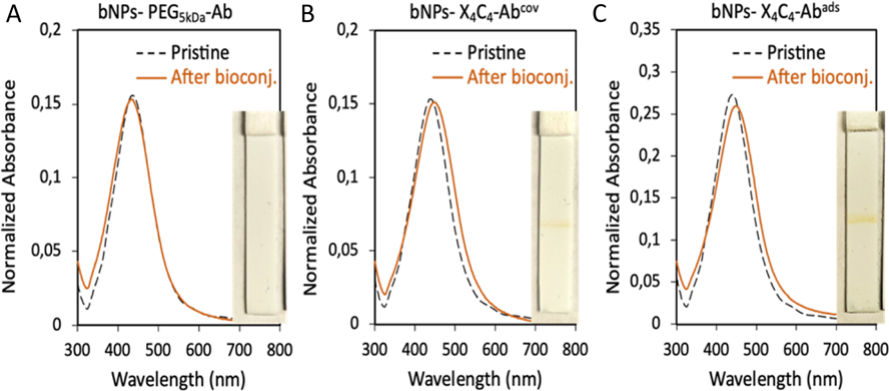
UV-vis spectra of bNPs before and after antibody bioconjugation and picture of the protein A/G strip used to assess the presence of antibodies for (A) αCD44 covalently grafted on bNPs-PEG_5kDa_, (B) αCD44 covalently grafted on bNPs-X_4_C_4_ and (C) αCD44 adsorbed on bNPs-X_4_C_4_.

The bNPs, gNPs and yNPs either coated by X_4_C_4_ or the thiolated PEG were bioconjugated with αCD44 or αHer2 antibodies (Ab) through classical peptide coupling chemistry using EDC and sulfo-NHS. The pH of the peptide coupling protocol had to be optimized for each antibody in order to ensure colloidal stability throughout the process: αHer2 Ab coupling was achieved at pH 8, while αCD44 Ab bio-conjugation was performed at pH 7. To ascertain the necessity of covalently attaching the biomolecules to the NPs, we also explored a faster and simpler alternative for bioconjugation with bNPs by performing one-step antibody adsorption. All batches of bioconjugated NPs presented sharp and intense extinction spectra, indicating no significant aggregation of the NPs upon bioconjugation (see Fig. 3 for bNPs). In all cases, the presence of antibodies was assessed using a protein A/G lateral flow assay (LFA), which relies on the high affinity of protein A/G for the heavy chains of antibodies. The interaction between the antibodies bound to the NP surface and the protein A/G deposited on the test line, leads to the immobilization of the nanoparticles on the test line, resulting in a positive, coloured line that displays the transmitted colour of the NPs (see the ESI† for more details). Bioconjugation of the various Abs was successfully achieved on both yNPs-X_4_C_4_ and yNPs-PEG_5kDa_, as attested by the positive red line visible on the LFA strips (Fig. S4†). However, in the case of bNPs and gNPs, the protein A/G LFA yielded a positive result only when the antibody was conjugated to the X_4_C_4_ coating, either *via* covalent bonding or adsorption (Fig. 3 and S4†). The absence of a coloured line with the bNPs and gNPs PEG_5kDa_ suggested the absence of conjugated antibodies.

Additionally, in contrast to NPs-X_4_C_4_, no redshift of the plasmon peak of these colloids was observed after the antibody bioconjugation step with NPs-PEG_5kDa_. To investigate whether this differential behaviour between the 100 nm AuNPs (yNPs) and the smaller Au:Ag alloy NPs (gNPs and bNPs) was due to the size or the intrinsic composition of the NPs, 60 nm AuNPs and AgNPs were also coated with the thiolated PEG_5kDa_ ligand and then bioconjugated as described above (Fig. S5 and Table S2†). A low mAb conjugation rate was also observed for 60 nm AuNPs and AgNPs, as indicated by the absence of a line on the LFA strip. This additional result suggests that the size and curvature of the NPs might affect the low accessibility of the carboxyl groups from the PEG chains, resulting in low Ab grafting efficiency.

### Protein detection in cells

**Fig. 4:**
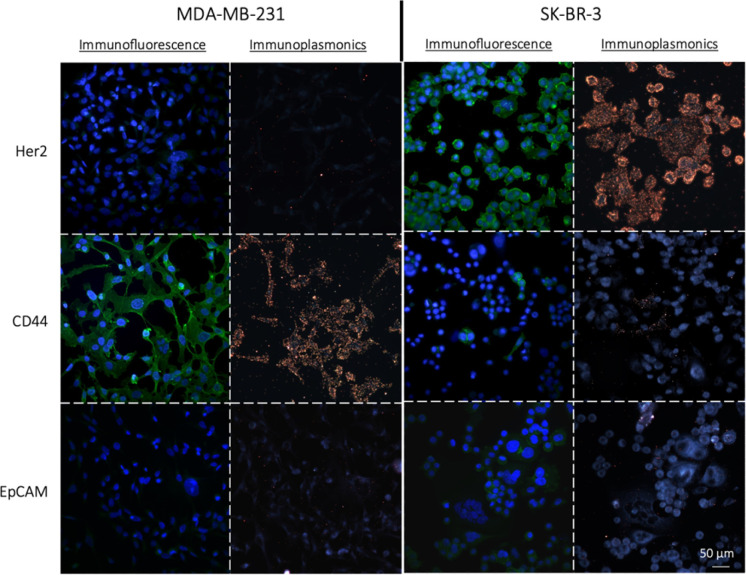
Immunofluorescence (protein of interest in green AF488, nuclei in blue, DAPI) and immunoplasmonics (yellow anti-protein NPs, yNPs-PEG_5kDa_-Ab) images of proteins Her2, CD44 and EpCAM in MDA-MB-231 and SK-BR-3 cell lines showing the equivalence between the results obtained with these two techniques. The images were acquired on an inverted Ti-Eclipse microscope (Nikon), a 40X-0.6NA air objective and using the epi-illumination arm (IF) or custom lateral illumination (IP).

To assess the effectiveness of the NPs-X_4_C_4_-Ab and NPs-PEG_5kDa_-Ab as immunoplasmonics protein tags in biological samples, we performed a one-step immunolabelling procedure with two breast cancer cell lines, MDA-MB-231 and SK-BR-3, which exhibit different levels of CD44, Her2 and EpCAM protein expressions. The protocol was adapted from the standard direct immunofluorescence method and involved a blocking step, followed by a one-hour incubation with NPs-Ab, and a washing step. As a control for the quantification of protein expression in the chosen cell lines, we employed a standard indirect IF procedure using the Abs that were used for bioconjugation and secondary antibodies labelled with AF488 (green fluorescence). The results from IF analysis revealed distinct patterns of protein expression in the SK-BR-3 and MDA-MB-231 cells for the three markers of interest (Fig. 4). Specifically, the SK-BR-3 cells displayed a strong expression of the Her2 protein whereas the MDA-MB-231 cells showed minimal expression. Additionally, our control experiments highlighted a unique behaviour within the SK-BR-3 cell population. Although the majority of these cells showed negligible expression of the CD44 protein, a small fraction demonstrated pronounced overexpression. In contrast, the MDA-MB-231 cells exhibited a strong homogeneous expression of the CD44 protein. Both cell lines weakly express the EpCAM protein, with a slightly higher expression level for the SK-BR-3 cell line. The full immunofluorescence data, including the negative controls with the secondary antibodies only, are included in the ESI (Fig. S6).†

First, using yNPs-PEG_5kDa_-Ab as protein labels, a strong correlation was observed between IP and indirect IF for both cell lines(Fig. 4). The staining of the cellular cytoplasm with Wheat Germ Agglutinin (WGA), along with the negligible binding of NPs to the glass substrate confirmed the co-localization of the NPs with the cell membrane, indicating a good specificity of the obtained yNPs-PEG_5kDa_-Ab (Fig. S7†). For further validation of the specificity of yNPs-PEG_5kDa_-αCD44, we conducted a dual-mode immunolabelling by applying both immunoplasmonics and immunofluorescence methods to the same sample of SK-BR-3 cells (Fig. S8†). This allowed us to confirm the equivalence of the results obtained with the two staining techniques, providing additional evidence for the reliability of immunoplasmonics. Similar results were obtained with yNPs-X_4_C_4_-Ab.

After confirming the potential of functionalized NPs as protein reporters, we applied the same procedure to bNPs coated with either PEG_5kDa_ or X_4_C_4_ and further bioconjugated to antibodies through EDC/sulfo-NHS chemistry (NPs-PEG_5kDa_-Ab and NPs-X_4_C_4_-Ab^cov^) or adsorption (NPs-X_4_C_4_-Ab^ads^). As expected, no significant cell or substrate binding was observed for bNPs-PEG_5kDa_-αCD44 (Fig. S9A†), in line with the LFA results (*vide supra*). For bNPs-X_4_C_4_-αCD44^ads^, we mostly observed non-specific attachment on the substrate, and few NPs on cells (Fig. S9B†), which was unexpected given the positive result from the LFA. This discrepancy might be due to an unfavorable orientation of the adsorbed Ab on the surface; however, further investigation is warranted to better understand this result. In contrast, bNPs-X_4_C_4_-αCD44^cov^ led to the expected heterogeneous binding with the SK-BR-3 cells (Fig. S9C†). Covalent grafting of antibodies on the NPs-X_4_C_4_ was therefore chosen as the preferred technique for NPs bioconjugation and was used for the rest of the study.

**Fig. 5:**
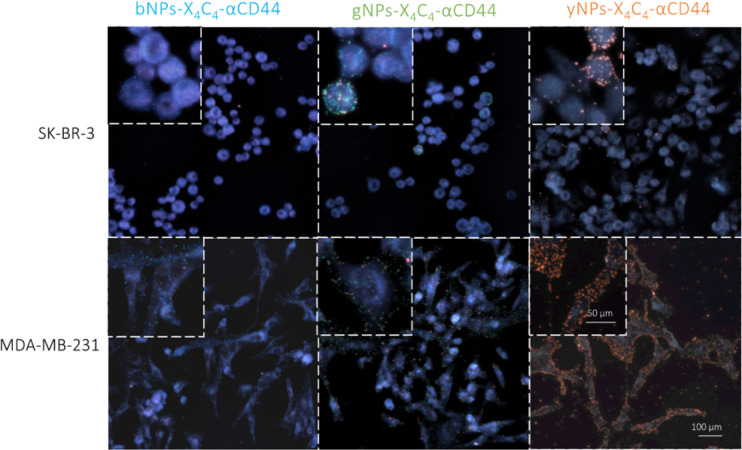
Immunoplasmonics images showing NPs-X_4_C_4_-αCD44^cov^ of various types used for CD44 protein detection on SK-BR-3 (top row) and MDA-MB-231 (bottom row) cell lines. For the SK-BR-3 cell line (top row), the insets show the heterogeneous attachment of NPs-X_4_C_4_-αCD44^cov^, which correlates with the CD44 expression found with immunofluorescence.

Next, we tested all types of NP-X_4_C_4_-Abs^cov^ on the two cell lines to validate their specificity. Fig. 5 shows the CD44 staining results on both cell lines using different colours of NPs, illustrating the tunability of the technique and the reliability of the results. Consistent results were also obtained with αHer2 antibodies (Fig. S10†). Upon initial examination, the optical properties of the various NP types were largely preserved during incubation with cells, and the samples prepared with a single NP type were readily distinguishable. These single-NP-type samples were later used as controls for the training of the automated classification software.

### Application to multiplexed immunolabelling on breast cancer cell lines

**Fig. 6:**
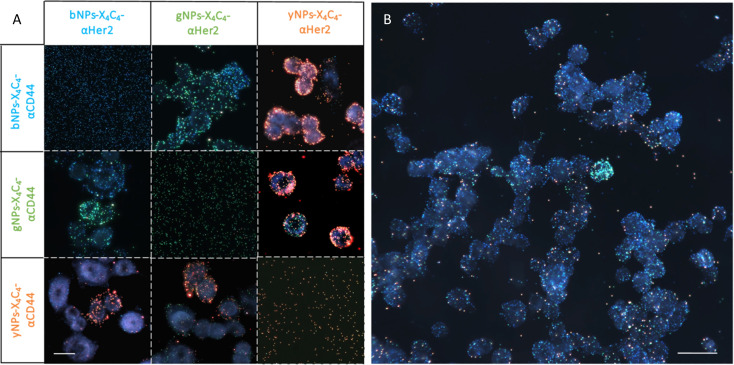
(A) Immunoplasmonics images showing the double detection of Her2 and CD44 proteins on the SK-BR-3 cell line using various NP types. The images on the diagonal display the corresponding NPs on a glass substrate. Scale bar is 25 μm in all images. (B) Triple detection of Her2, CD44 and EpCAM on the SK-BR-3 cell line using respectively bNPs-X_4_C_4_-αHer2, gNPs-X_4_C_4_-αCD44 and yNPs-X_4_C_4_-αEpCAM. Scale bar is 50 μm.

After confirming the feasibility of antibody grafting on each type of NPs and the ability of these hybrid nanoconjugates to target specific receptors at the surface of cells, we used various combinations of these bioconjugated NPs for dual immunolabelling on both cell lines. The results for the SK-BR-3 cell line are depicted in Fig. 6A. For clarity, the images were cropped to emphasize cases where a cell over-expresses CD44 and under-expresses Her2 compared to its neighboring cells. This phenomenon was corroborated through dual immunofluorescence, even though some cells expressed both receptor types in similar proportions, as observed through both techniques. Examples of dual immunofluorescence assays are available in the ESI (Fig. S11).† Additionally, we achieved consistent plasmonic immunolabelling on the MDA-MB-231 cell line, as shown in the ESI (Fig. S12).† Similarly, immunolabelling of three markers was achieved on both cell lines, using bNPs, gNPs, and yNPs to target Her2, CD44, and EpCAM proteins, respectively. The comparison between the two cell lines is provided in the ESI (Fig. S13),† validating the possibility of performing multiplexed detection of up to three membrane receptors (Fig. 6B).

### Image analysis and automated classification of NPs

**Fig. 7:**
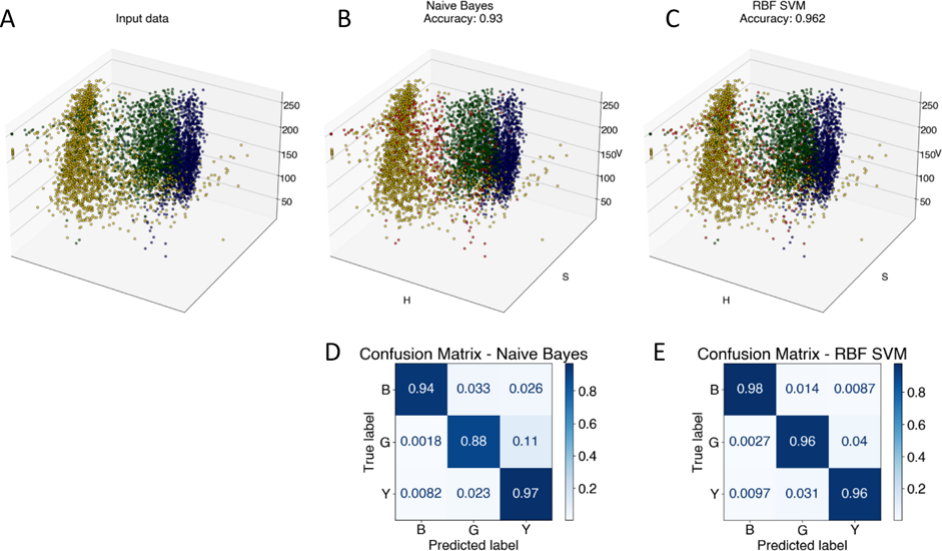
(A) – Input data. Each dot represents a single NP in the HSV color space. The color of the dot represents their class. (B) – result of the classification on the validation dataset using a naïve Bayes classifier. The red dots correspond to misclassified NPs. (C) – result of the classification using a Support Vector Machine (SVM) with a Radial Basis Function (RBF) as a kernel. (D and E) – are the corresponding confusion matrixes, normalized according to the real number of NPs of each class in the testing set.

To advance the use of bio-conjugated NPs as protein reporters, we developed an automated classification and tested various standard supervised classification techniques. The software is publicly available on GitHub.§§The software is available at: https://github.com/cdarviot/Classifier.git. The best results were obtained using a Support Vector Machine (SVM) with a Radial Basis Function (RBF) kernel on normalized Hue, Saturation and Value (HSV) signals extracted from the NPs, resulting in three features per NP. First, each RGB image is transferred to the HSV colour space. The HSV colour space was selected as it enables a better separation of the colour and saturation from the brightness, making the colour identification more resilient to variations in lighting and illumination. Then, an algorithm based on local maxima detection locates each NP present in the image. The average signals from a 5 × 5 pixels region of interest (ROI) around each NP location are extracted, resulting in a feature vector composed of the mean H, S and V values associated with each NP detected. Fig. S14† illustrates the different representations of the NPs signals in RGB or HSV colour space. A dataset composed of ten thousand labelled NPs was normalized, split into a training set, used to train the model, and a validation set, used to assess the algorithmic performances. Fig. 7 shows the input data (Fig. 7A), considered as the ground truth, the classification of these data using a Naïve Gaussian Bayes classifier (Fig. 7B), and the classification using the supervised SVM with an RBF kernel (Fig. 7C). A way to better encompass the performances of classification algorithms is to use the confusion matrix, which compares the ground truth (true label) to the classification output (predicted label). The confusion matrixes for the two classifiers are also depicted in Fig. 7D and E.

**Fig. 8:**
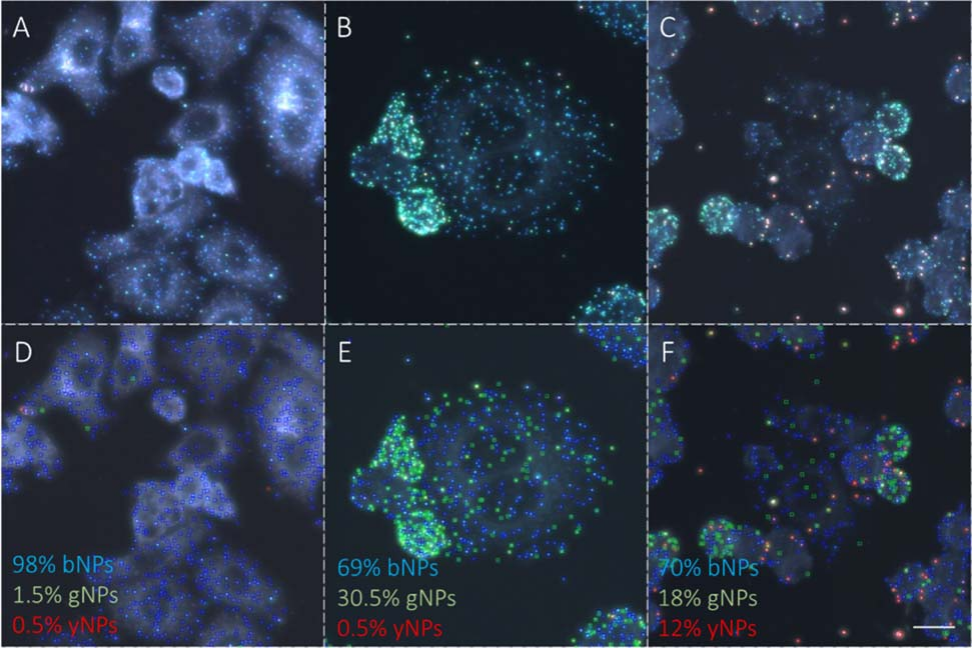
Example of automated classification results on SK-BR-3 cells incubated with (A) bNPs-X_4_C_4_-αHer2 (2 × 10^9^ NPs/mL), (B) – a mixture of bNPs-X_4_C_4_-αHer2 (2 × 10^9^ NPs/mL) and gNPs-X_4_C_4_-αCD44 (2 × 10^9^ NPs/mL), (C) – a mixture of bNPs-X_4_C_4_-αHer2 (2 × 10^9^ NPs/mL), gNPs-X_4_C_4_-αCD44 (2 × 10^9^ NPs/mL) and yNPs-X_4_C_4_-αEpCAM (2 × 10^9^ NPs/mL). (D–F): corresponding labels automatically generated by the classifier. Scale bar is 50 μm.

Then, we used the trained classifier to count the number of nanoparticles of each type in the various multiplexed samples, as shown in Fig. 8A shows the results of the classification performed on a new sample prepared with bNPs-X_4_C_4_-αHer2 only. The algorithm classified 98% of the NPs as blue, which is the expected answer given that we have incubated the cells with blue NPs only. We can see that among the NPs that were misclassified, some were considered green (1.5%) or yellow (0.5%). For each class *k*, the error in classification can be measured as follows:1
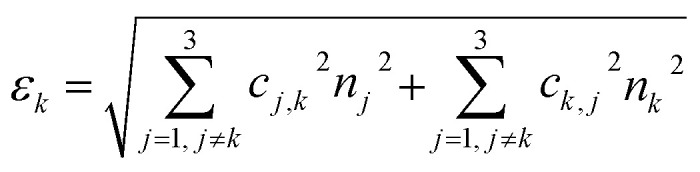
where the first terms represent NPs from other classes classified as class *k* (false positive) and the second term NPs from class *k* that were classified as other NP types (false negative). In the eqn (1), *c*_*i*,*j*_ are the coefficients from the confusion matrix, and *n*_*i*_ the number of particles from class *i*. To calculate the total classification error, we used the confusion matrix that was obtained after the training (Fig. 7E). For instance, in the entire image from which we extracted Fig. 8C, we counted 3107 ± 42 bNPs, 783 ± 29 gNPs, and 553 ± 31 yNPs, which is consistent with the expression levels of the proteins in the cell line, as validated with IF.

## Discussion

We have successfully confirmed the potential of plasmonic nanoparticles as protein reporters. While the use of HS-PEG_5kDa_-COOH as a surface ligand appeared suitable for the bioconjugation of antibodies with 100 nm AuNPs, the NPs coating with calix[4]arene X_4_C_4_ proved to be a much more versatile strategy for the bioconjugation of antibodies with nanoparticles of various compositions and sizes. The stability of NPs-PEG_5kDa_ in PBS clearly indicates the presence of the PEG layer at their surface for all types of NPs. However, both LFAs and incubation with cells have shown poor antibody coupling efficiency to our smaller NPs using the PEG_5kDa_ linker. The absence of reactivity of the terminal COOH groups with amino groups upon peptide coupling conditions could be due to several reasons. It is known that for a given molecular weight, the grafting density achieved is lower for smaller nanoparticles.^28^ Hence, for the smaller nanoparticles, the greater radius of curvature could lead to an increasing distance between two PEG strands, allowing the bending of their tips in a mushroom-like configuration, as reported previously for PEG_5kDa_ on 60 nm AuNPs.^29^ This arrangement could potentially heighten steric hindrance around the carboxyl group, preventing an efficient bioconjugation. Another issue regarding the PEGylation of NPs containing silver was the observed desorption with time, leading to a significant loss in colloidal stability. While the strategy adopted for the PEGylation leverages the adsorption of thiols on gold and silver surfaces, the mechanism of grafting of X_4_C_4_ on metal surfaces involves the reduction of its aryl diazonium units into the corresponding aryl radicals, which subsequently react with the metal surface to form strong and irreversible covalent metal–C bonds.^26^ X_4_C_4_ being equipped with four diazonium groups, each macrocycle can form up to four metal–C bonds with the surface, resulting in an increased density of anchoring points and much more robust coating compared to the use of simple aryl diazonium salts or thiolated ligands.^23^ The latter is generally considered as covalent bond but, however, is still subject to debate in literature. Previous studies determined a density of 1.5 X_4_C_4_/nm^2^ for AuNPs of 15 nm and 0.53 X_4_C_4_/nm^2^ for flat surfaces.^30,31^ Therefore, we can assume an intermediate value of grafting density for NPs size ranging from 60 to 100 nm. Overall, the X_4_C_4_ coating has shown superior performances in terms of stability and bioconjugation than the PEG_5kDa_ coating.

The preparation of the samples containing a single NP type also gives an insight into the maximal precision that could be reached with the technique, considering the experimental conditions studied in this work. Indeed, although they represented less than 5% of the NPs detected, few NPs did not display the expected colour. Various factors could explain the discrepancies, ranging from disparity in the synthesis to the formation of clusters during the bioconjugation process or the incubation with the cells. This issue could be mitigated by investigating filtering steps using either a mechanical filter or centrifugation steps prior incubation with the cells. Regarding the multiplexed detection of proteins, it is noteworthy that the incubation of the multiple NP types was conducted in one step, resulting in a significant gain of time as compared to subsequent incubations. Importantly, mixing the various NP types did not increase the aggregation levels of the colloids, leading to highly distinguishable labelling of each protein type. Once again, the comparison with immunofluorescence has reaffirmed that the observed NP attachment aligns with the protein expressions of the studied cell lines. Yet, the immunoplasmonic technique exhibits greater versatility compared to multiplexed immunofluorescence, as it is unaffected by antibody host constraints which requires making some compromises when elaborating a multiplexed antibody panel. Moreover, it is not sensitive to channel bleed-through, which typically necessitates extensive calibration for intensity quantification. Although all the prepared NPs-X_4_C_4_-Ab have shown the expected behaviour, it was noted that direct visual examination of the samples was challenging for some NPs combinations. For instance, the chosen yNPs display a much higher scattering level than the bNPs. In addition, within the MDA-MB-231 cell samples, discerning the sparse bNPs-X_4_C_4_-αHer2 among the numerous yNPs-X_4_C_4_-αCD44 was particularly challenging without image analysis. Conversely, one can leverage the great brightness of yNPs to detect rare antigens, as demonstrated in our ability to detect the EpCAM protein in both cell lines and the Her2 protein in MDA-MB-231 cells. Compared to more complex techniques, such as immunofluorescence or Raman spectroscopy, a very simple apparatus can be affixed to any microscope and a standard RGB image is only needed to enable the automated analysis. As for the automated detection of NPs, the use of a simple supervised training algorithm for classification such as a Support Vector Machine has helped to gain in accuracy compared to naïve classification based on Bayes filtering. The software developed in this study will prove invaluable for future research endeavours, particularly in the calibration of the developed technique, with a notable focus on advancing protein quantification using these nanoparticles.

## Conclusions

We have developed an effective methodology for grafting antibodies onto a wide range of plasmonic nanoparticles, which offers essential advantages for immunolabeling while preserving their distinct optical properties. Furthermore, in comparison with the classical coating approach using thiolated PEG ligands, we have demonstrated the superiority of the calix[4]arene coating for the formation of ultrastable and bioconjugable plasmonic NPs of various sizes and compositions. The utility of these calix[4]arene-based nanoparticles has been corroborated through their efficient use as multiplexed optical tags for the detection of a variety of membrane proteins. Additionally, a sophisticated software tool has been developed for automated nanoparticle quantification in biological samples based on their class, further emphasizing the practical applications of immunoplasmonic in clinical settings. While triple multiplexing has been shown in this work, using NPs presenting other plasmonic peaks coupled with potentially hyperspectral imaging may open up the possibility of offering a higher level of multiplexing to biologists and pathologists.

## Conflicts of interest

There are no conflicts to declare.

## Supplementary Material

NA-006-D4NA00052H-s001
