# Supplementary material for: Multiplexed immunolabelling of cancer using bioconjugated plasmonic gold–silver alloy nanoparticles
Source: Nanoscale Adv. 2024 Jul 3;6(17):4385–93. doi: 10.1039/d4na00052h (PMC11334976; doi:10.1039/d4na00052h)
Supplement: NA-006-D4NA00052H-s001 [file NA-006-D4NA00052H-s001.pdf]

Electronic Supplementary Material (ESI) for Nanoscale Advances.
This journal is © The Royal Society of Chemistry 2024

Multiplexed immunolabelling of cancer using bioconjugated plasmonic gold-silver alloy nanoparticles.

Authors :

C. Darviot[1,2], B. Gosselin[3], F. Martin[1], S. Patskovsky[1], I. Jabin[3], G. Bruylants[4], D. Trudel[2]* and M. Meunier[1]*.

[1] Polytechnique Montréal
[2] Centre Hospitalier de l'Université de Montréal
[3] Université libre de Bruxelles, LCO
[4] Université libre de Bruxelles, EMNS

* Authors contributed equally

Synthesis of alloy nanoparticles

Materials

Gold (III) chloride trihydrate ($HAuCl_4 \cdot 3H_2O$, ≥99.9%), was purchased from Sigma-Aldrich. Silver Nitrate ($AgNO_3$, ≥99.9995%), was purchased from Alfa Aesar. Sodium citrate dihydrate (NaCit) ($Na_3C_6H_5O_7 \cdot 2H_2O$, 99.0%) was purchased from Alfa Aesar. The 18 MΩ · cm deionized (DI) water is taken from an EMD Millipore Direct-Q 3 UV ultrapure water purification system. Glassware is cleaned with aqua regia solution before NPs synthesis to remove metal residues and thoroughly rinsed with DI water.

Methods

Spherical Au:Ag NPs were synthesized according to a previously reported method [1,2]. Briefly, synthesized AuNPs seeds are used for subsequent seeded growth of size- and composition-controlled Au:Ag alloys by coreduction of gold and silver salts. The amount of metal precursor ions added during a given growth step determines the final size of the synthesized Au:Ag NPs. The ratio between the amount of Au and Ag metal precursor ions added dictates the final composition of the synthesized Au:Ag NPs. During seeded growth steps, the reducing agent NaCit is added before the metal ion solutions to mediate solution pH and slow reduction speed resulting is increased NPs homogeneity. Furthermore, the free metal ion concentration is controlled by performing multiple injections of metallic salt solutions to avoid secondary NPs nucleation. Additionally, the chemical reduction rate of metal ions is regulated through dynamic temperature control to insure NPs shape monodispersity.

AuNPs seeds are synthesized using the Turkevich method[3]. In quick succession, 300 µL of 30 mM $HAuCl_4$ solution and 200 µL of 170 mM NaCit are added to 30 mL of boiling DI water in an Erlenmeyer flask under vigorous magnetic stirring. Boiling and stirring are maintained for 15 minutes. The solution is then cooled to room temperature. After the solution has reached room temperature, the volume is readjusted to 30 mL by adding DI water to account for evaporation, thus ensuring the atomic concentration of gold is 300 µM. The size of the synthesized AuNPs seeds is 15 ± 1 nm in diameter.

Multiple seeded growth steps were subsequently performed to achieve the final desired Au:Ag size. The AuNPs seeds are used for the first growth step. The Au:Ag alloys synthesized during a given growth step are used as seed for the subsequent growth steps. Au:Ag 10:90 were synthesized in 2 steps (32 nm to 50 nm), and the Au:Ag 50:50 NPs were synthesized in 3 steps (32 nm to 50 nm to 60 nm).
For the synthesis of Au:Ag 10:90 NPs measuring 50 nm, 3 mL of AuNPs seeds are added to 57 mL of 90°C DI water under vigorous magnetic stirring in a three-necked flask, set in a silicon oil heating bath and connected to a reflux condenser. Then, a total of 540 µL of 170 mM NaCit, 27 µL of 30 mM $HAuCl_4$ and 243 µL of 30 mM $AgNO_3$, are added in 10 successive injections every 6 minutes. After all ten injections, the temperature of the heating bath is raised to 100°C and maintained for 1 hour. The resulting NPs are spherical Au:Ag 10:90 NPs with a diameter of 32 ± 2 nm. A second growth step is

performed using Au:Ag 10:90 NPs 32 nm as seeds to obtain the desired Au:Ag 10:90 NPs with a diameter of 50.8 ± 6.6 nm. The synthesis of Au:Ag 50:50 60 nm is achieved with 3 growth steps following a similar protocol with adjustments to the ratio between the amount of Au and Ag metal precursor ions. Reagent volumes used for the various synthesis steps are detailed in table XX and XY.

**GROWTH STEP**	**SEEDS**	**SEEDS VOLUME (ML)**	**NACIT 170 MM VOLUME (µL)**	**HAUCL$_4$ 30 MM VOLUME (µL)**	**AGNO$_3$ 30 MM VOLUME (µL)**	**RESULT**
**1**	AuNPs 15 nm	3	540	27	243	Au:Ag 10:90 32 nm
**2**	Au:Ag 10:90 32 nm	15	450	22.5	202.5	Au:Ag 10:90 50 nm

Growth Step	*Seeds*	*Seeds Volume (mL)*	*NaCit 170 mM Volume (µL)*	*HAuCl$_4$ 30 mM Volume (µL)*	*AgNO$_3$ 30 mM Volume (µL)*	*Result*
1	AuNPs 15 nm	3	540	135	135	Au:Ag 50:50 32 nm
2	Au:Ag 50:50 32 nm	15	450	112.50	112.50	Au:Ag 50:50 50 nm
3	Au:Ag 50:50 50 nm	20.69	393.10	98.28	98.28	Au:Ag 50:50 60 nm

Coating of alloy nanoparticles: SH-PEG_{5kDa}-COOH vs calixarene

Full name	*Short name*	*TEM Ø*	*Hydrodynamic Ø*	*Transmitted/scattered colour*
Au:Ag 10:90 50nm	bNPs	50,8 ± 6,6	51,4 ± 0,5	Yellow/Blue
Au:Ag 50:50 63nm	gNPs	62,5 ± 3,5	70,7 ± 1,5	Orange/Green
Au 100nm	yNPs	104,8 ± 5,5	98,8 ± 0,7	Pink/Yellow

Table S1: Characteristics of the NPs

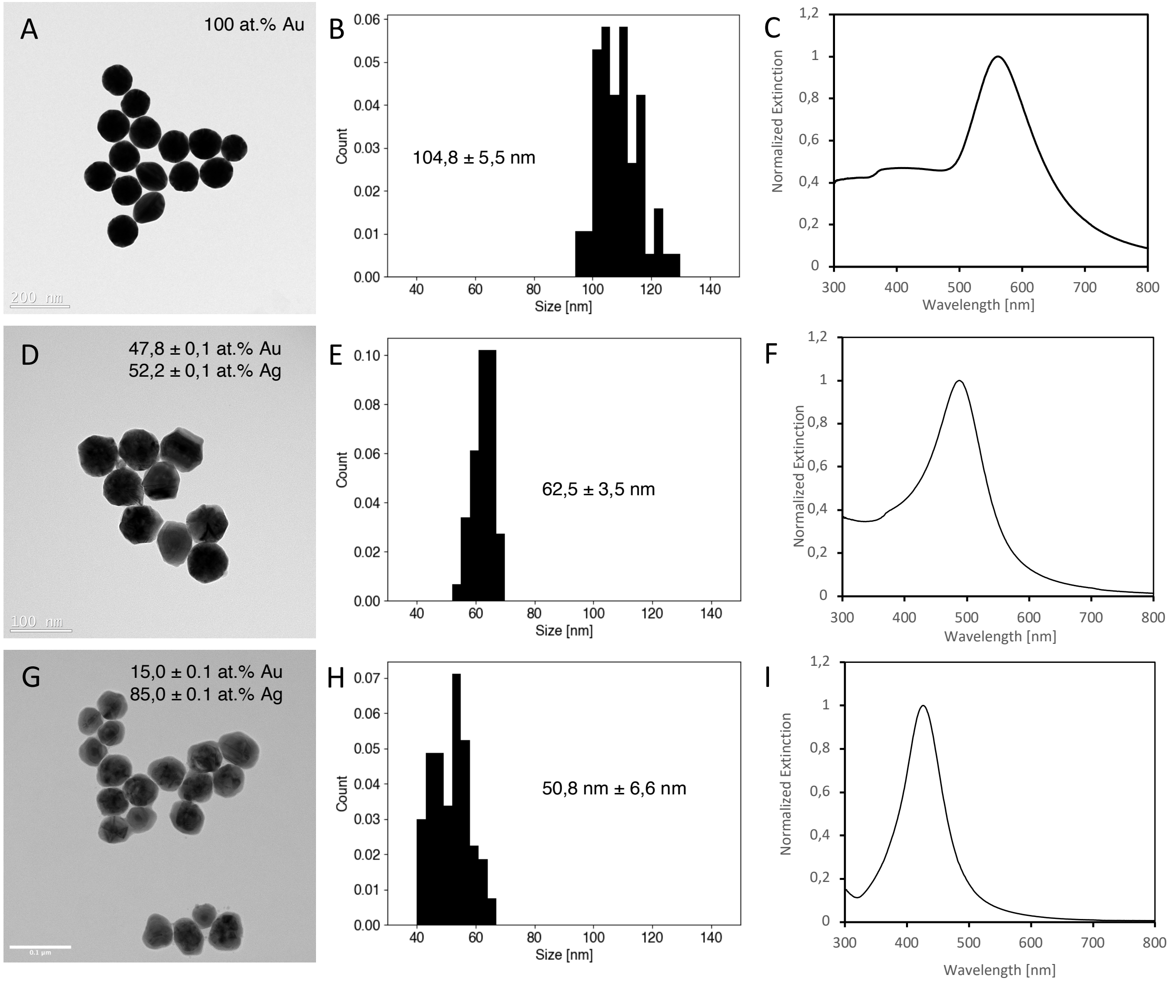

Figure S1. Characterization of bare NPs with TEM (A-D-G), measured size distributions (B-E-F) and UV-Vis spectroscopy (C-F-I) for the chosen NPs: 100nm AuNPs (A-B-C), 50:50 Au:Ag 63 nm NPs (D-E-F) and 10:90 Au:Ag 50 nm (G-H-I).

Coating of alloy nanoparticles: PEG vs calixarene

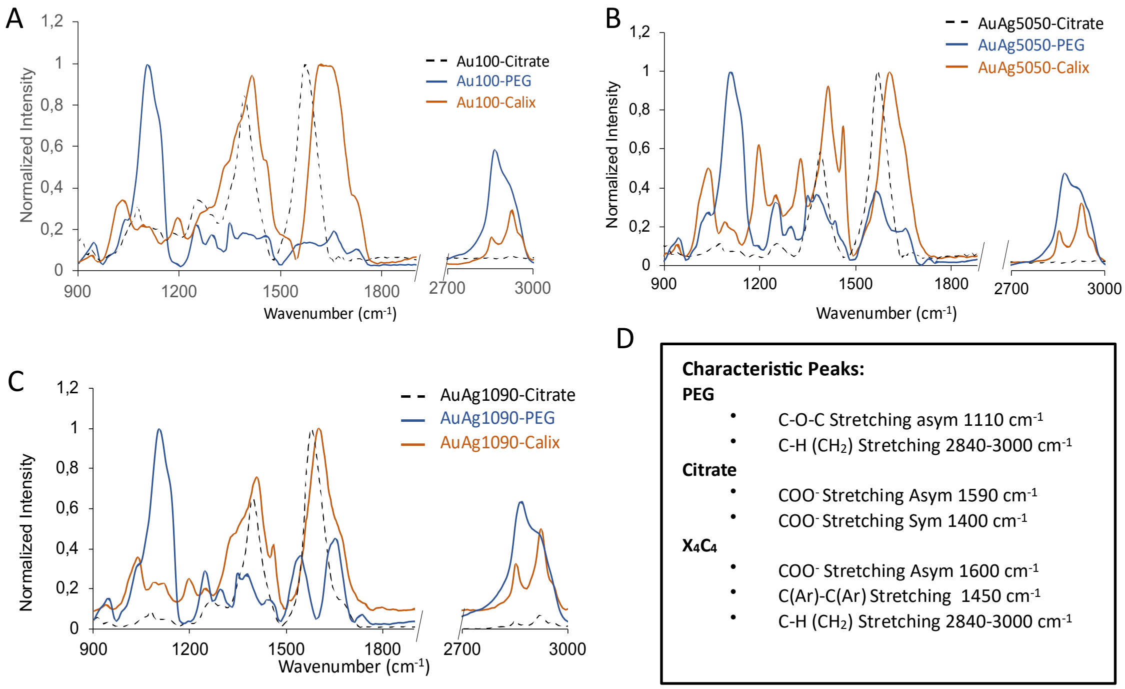

Figure S2: FTIR spectra of bare and functionalized NPs for A – yNPs, B – gNPs, C – bNPs and D – Summary of the main characteristic bands measured for each coating.

Bioconjugation using EDC/NHS cross-linking chemistry.

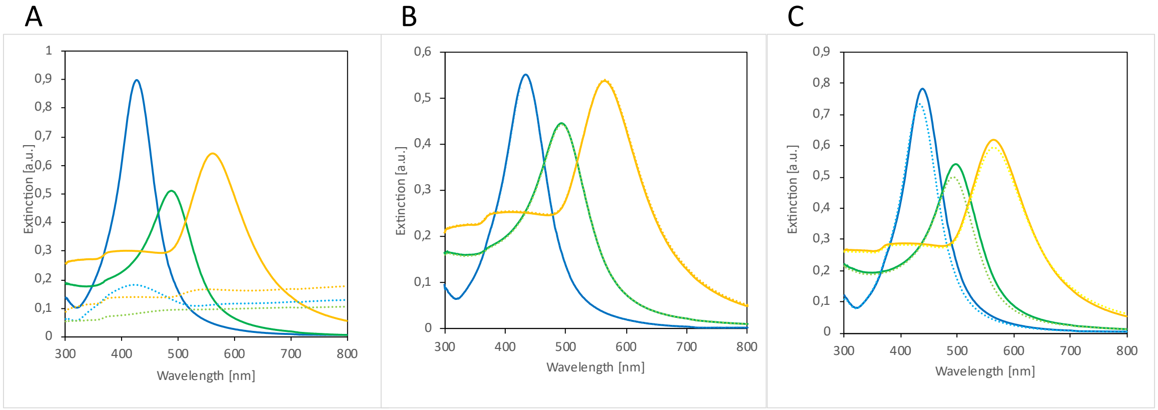

Figure S3. Stability of blue, green, and yellow colloidal nanoparticles in dH_2O (plain lines) or PBS 1X (dotted lines) for: A – citrate capped NPs; B – PEGylated NPs and C – NPs-X_4C_4.

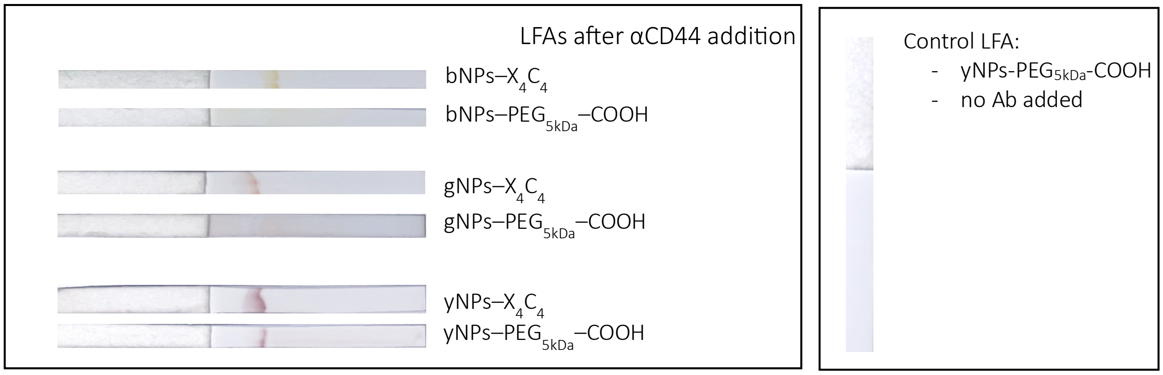

Figure S4: LFAs for αCD44 grafting on the various types of NPs. The color we see on the LFAs is the transmitted color and not the scattered color. Similar results were obtained with other Abs.

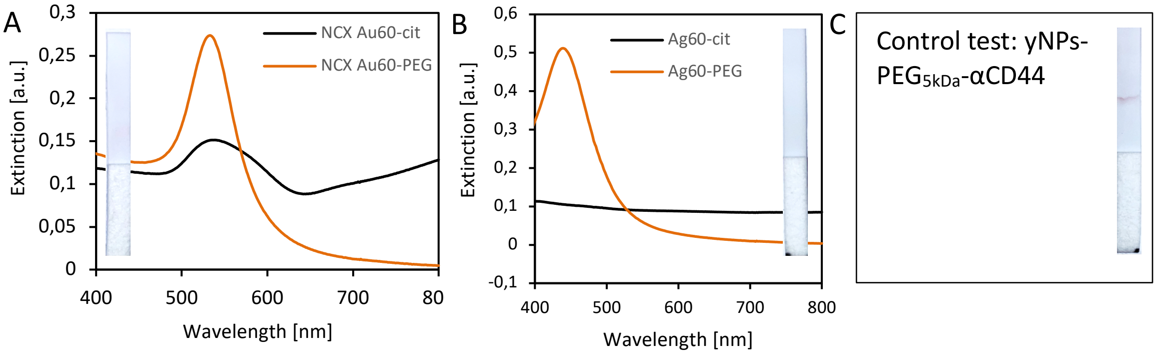

Figure S5: LFAs for the A) pure gold or B) pure silver 60 nm NPs and their stability in PBS 0,5X after 30min. C) is the LFA control with the bioconjugation of yNPs-PEG_{5kDa} performed at the with the same reagents as the other two.

	Bare	*PEG_{5kDa}*	*Δd_H*
Ag 60nm	63,8	91,8	28
Au 60nm	63,5	93,4	29,9

Table S2: Hydrodynamic diameters of 60nm pure Ag and pure gold NPs before and after adding SH-PEG_{5kDa}-COOH.

Protein detection in cells

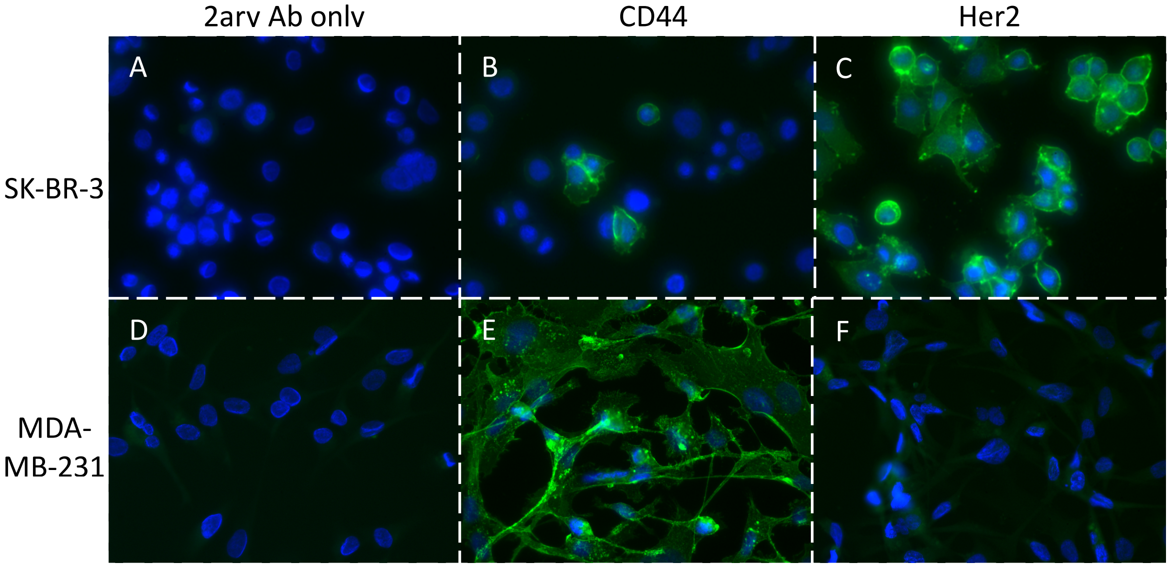

Figure S6: Immunofluorescence showing the expression of CD44 (B&E) and Her2 (C&F) for respectively the SK-BR-3 and the MD-MB-231 cells, as compared to the control, obtained with only the secondary antibody. The presence of CD44 is revealed through the green fluorescence (AF488) of secondary antibodies (2ary Ab). Nuclei are stained in blue with DAPI.

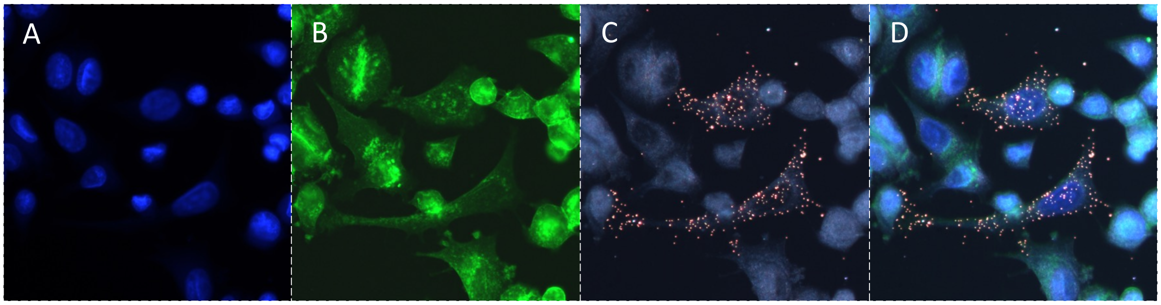

Figure S7: proof of good concordance of the membrane vs NPs attachment in SK-BR-3 cell line. A) DAPI staining showing the nuclei of the cells in blue; B) WGA staining showing in green (AF488) the cytoplasm of the cells; C) Immunoplasmonics staining towards CD44 detection; D) Composite image combining A, B and C.

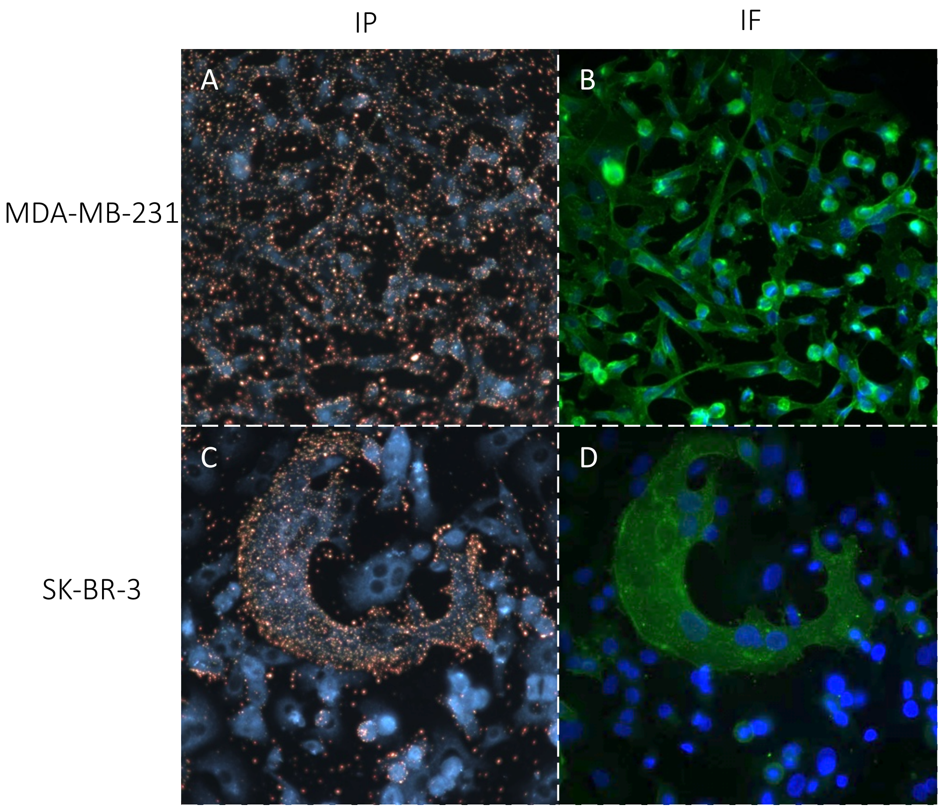

Figure S8: Double confirmation of the specificity of the detection of CD44 using IF and IP. Immunostaining of CD44 protein in MDA-MB-231 cell line with IP technique (A) and IF (B) in the same sample. Immunostaining of CD44 protein in SK-BR-3 cell line with IP technique (C) versus IF (D) in the same sample. In IF, the protein is revealed by the green fluorescence from secondary antibodies and the nuclei are stained in blue.

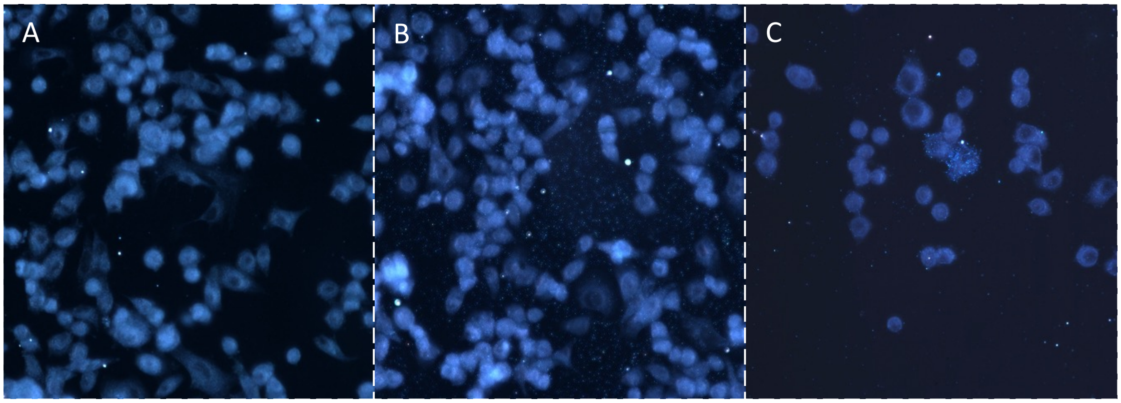

Figure S9: SK-BR-3 cells after incubation with A) the $NP_{Au:Ag\ 10:90}$-PEG_{5kDa}-αCD44, B) the $NP_{Au:Ag\ 10:90}$-C_4X_4-αCD44^{ads} and C) the $NP_{Au:Ag\ 10:90}$-C_4X_4-αCD44^{cov}. Scale bar is 50 µm.

Application to multiplexed immunolabelling on breast cancer cell lines.

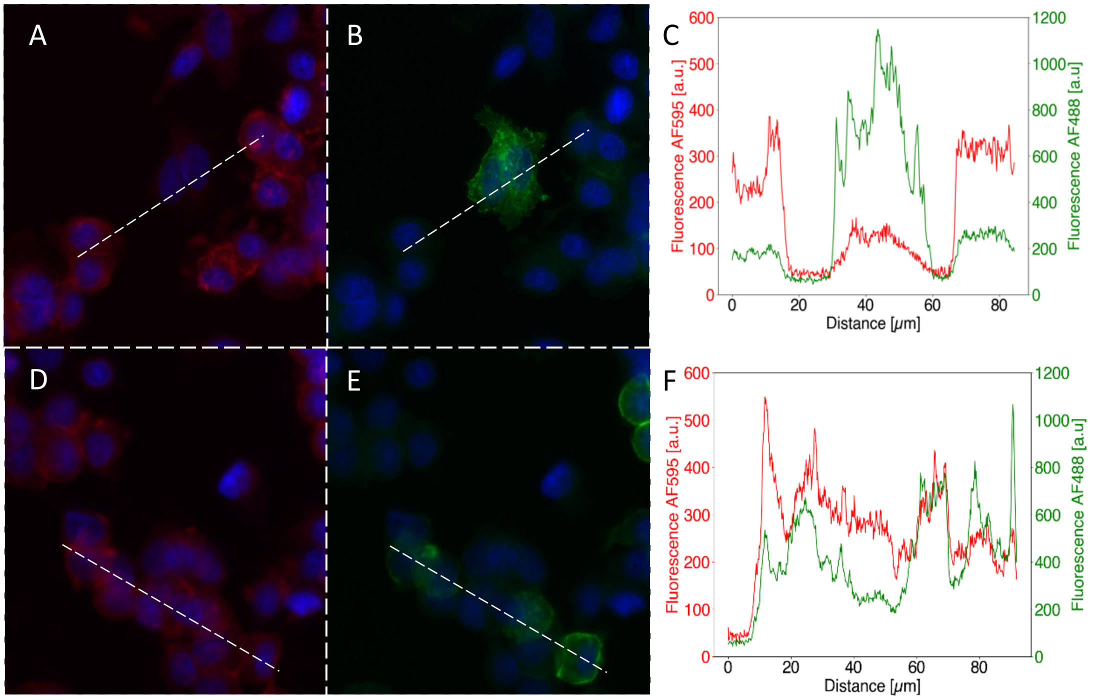

Figure S10: Multiplexed IF on SK-BR-3 cell line, using an anti-rabbit-AF495 (in red) to reveal Her2 and anti-rat-AF488 (green) to reveal CD44 protein. Nuclei are stained in blue with DAPI. Top panel (A & B) shows a case where a cell overexpressing CD44 also underexpresses Her2, C) is the corresponding intensity profile along the dashed line. Bottom panel (D & E) shows a case where there is no significant loss in Her2 expression for the cells overexpressing Her2, F) is the corresponding intensity profile along the dashed line. Both behaviours were also found using IP as a labelling technique.

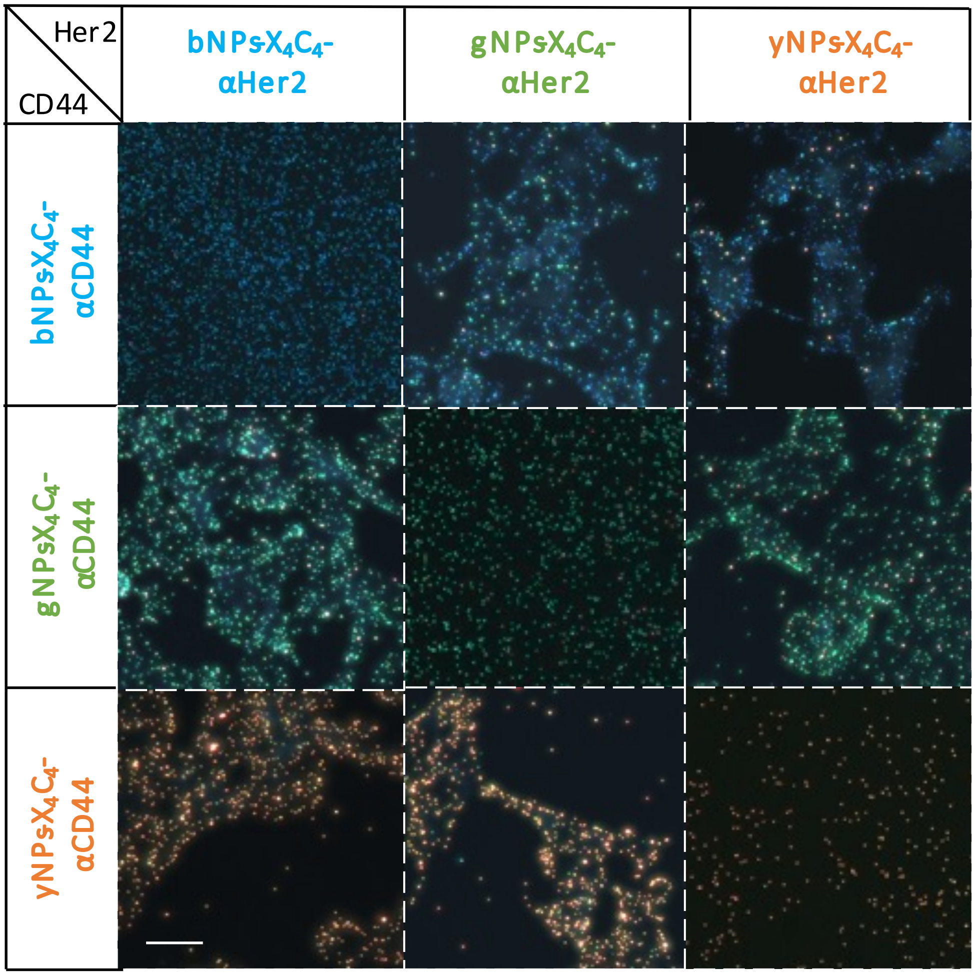

Figure S11: Dual detection on MDA-MB-231 cell line. As expected, Her2 being weakly expressed by the MDA-MB-231 cells, the cells display mostly the NPs detecting the CD44 proteins.

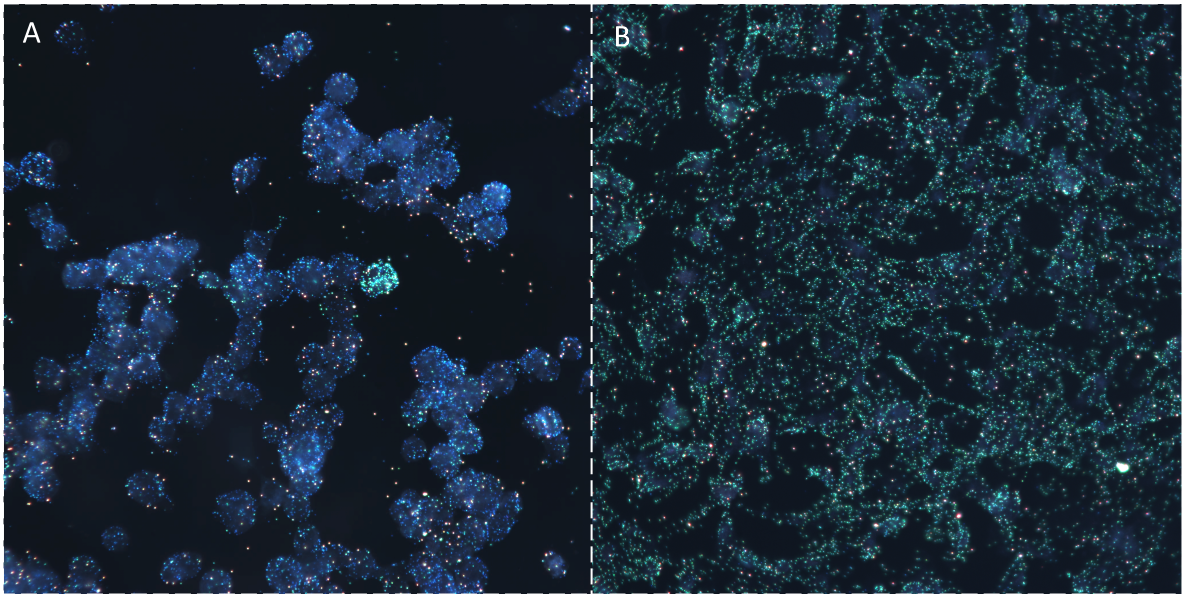

Figure S12: Triple detection of Her2, CD44 and EpCAM proteins in A) SK-BR-3 cell line; B) MDA-MB-231 cell line, using bNPs-X_4C_4-αHer2, gNPs- X_4C_4-αCD44 and yNPs- X_4C_4-αEpCAM

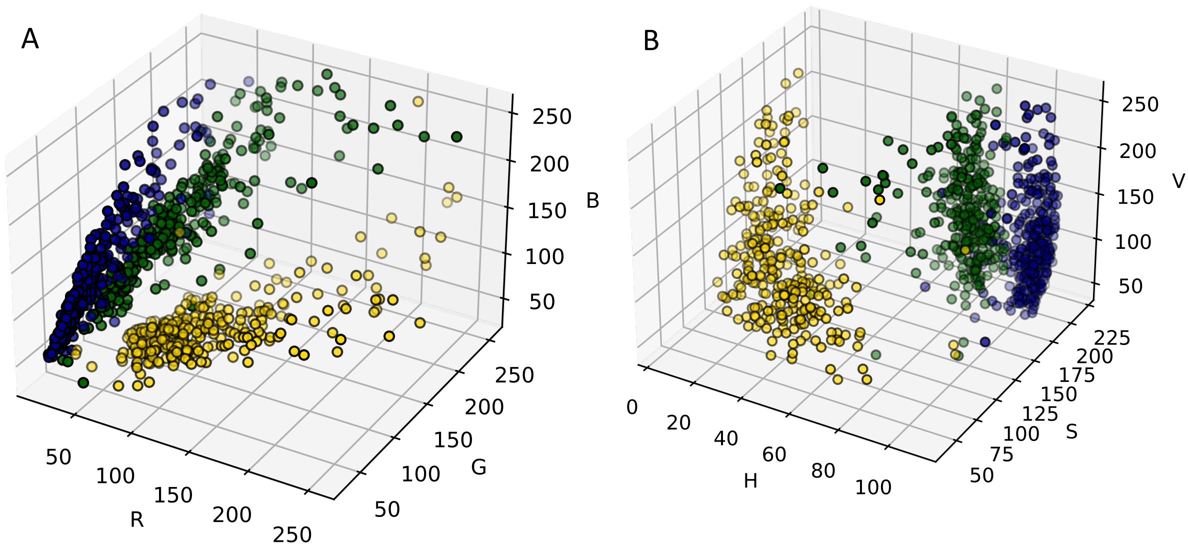

Figure S13: Extracted signals from the NPs as represented in A – the Red – Green – Blue (RGB) colour space, B – the Hue – Saturation – Value (HSV) colour space.

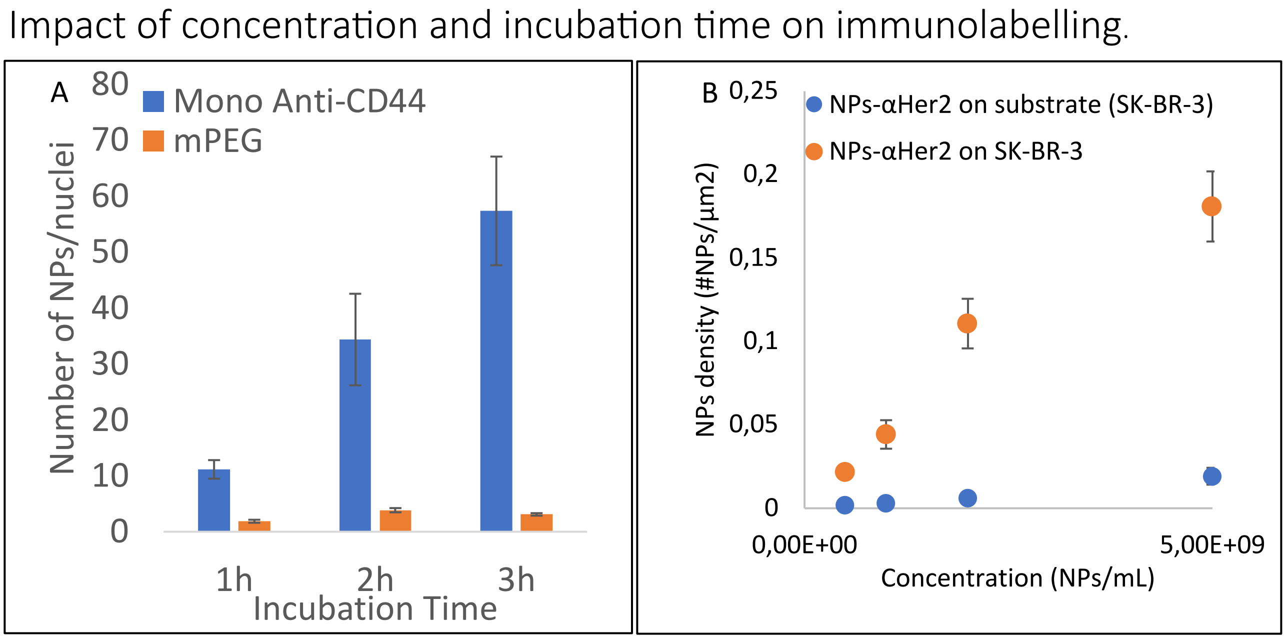

Figure S14 : A – Influence of the incubation time on NPs-anti-CD44 attachment on the MDA-MB-231 cells (C = 5.108 NPs/mL). mPEG NPs are used as negative control. B – Impact of NPs concentration on the attachment of NPs-anti-Her2 on the SK-BR-3 cells (incubation time 3h).

As for any immunolabelling technique, the concentration and incubation time have an impact on the outcome of the labelling. Fro practical reasons we have decide to set the time to 1h (similar to routine pathology immunolabelling) and concentration to 2E9 NPs/mL.
